# Can Molecular Classifications Help Tailor First-line Treatment of Metastatic Renal Cell Carcinoma? A Systematic Review of Available Models

**DOI:** 10.1016/j.euros.2022.11.006

**Published:** 2022-12-15

**Authors:** Idir Ouzaid, Nathalie Rioux-Leclercq, Zine-Eddine Khene, Karim Bensalah, Solène-Florence Kammerer-Jacquet

**Affiliations:** a Department of Pathology, University of Rennes, CHU Rennes, Inserm, EHESP, Irset (Institut de recherche en santé, environnement et travail)—UMR_S1085, Rennes, France; b Department of Urology, Bichat Claude Bernard Hospital, University of Paris, Paris, France; c Department of Urology, CHU de Rennes, Rennes, France

**Keywords:** Renal cell carcinoma, Metastases, Tyrosine kinase inhibitor, Immune check inhibitors, Angiogenesis, Immunotherapy, PD-L1, Biomarkers

## Abstract

**Context:**

The advent of immune check inhibitors (ICIs) has tremendously changed the prognosis of metastatic renal cell carcinoma (mRCC), adding an unseen substantial overall survival benefit. These agents could be administered alone or in combination with anti–vascular endothelial growth factor (anti-VEGF) therapies. So far, treatment allocation is based only on clinical stratification risk models.

**Objective:**

Herein, we aimed to report the different molecular classifications reported in the first-line treatment of mRCC and discuss the awaited clinical implications in terms of treatment selection.

**Evidence acquisition:**

Medline database as well as European Society for Medical Oncology (ESMO)/American Society of Clinical Oncology (ASCO) conference proceedings were searched to identify biomarker studies. Inclusion criteria comprised randomized and nonrandomized clinical trials that included patients treated in the first line of mRCC setting, patients treated with anti-VEGF therapies or ICIs, biological modeling, and available survival outcomes.

**Evidence synthesis:**

Four classification models were identified with subsequent clinical implications: Beuselinck model (34 gene signatures), IMmotion150, Hakimi, and JAVELIN 101 model. Tumor profiling shows distinct outcomes when treated with one or other combination. Patients are clustered into two gene signatures: angiogenic and proinflammatory (as per JAVELIN). The first is more likely to respond to therapy that includes anti-VEGF agents, while the best outcomes are obtained with an ICI combination with the second.

**Conclusions:**

The findings presented here were mostly derived from ancillary registered studies of new drugs in the setting of mRCC. Further validation is needed, which sets new paradigms for investigation in clinical research based on tumor biology for treatment allocation and not only on clinical stratification tools.

**Patient summary:**

First-line treatment of metastatic kidney includes immunotherapy alone or in combination with antiangiogenic therapy. However, clinical practice demonstrated that the “one treatment fits all” strategy might not be the best approach. In fact, recent studies showed that the addition of immunotherapy agents will not benefit all patients equally, and some still respond either equally to or better than anti–vascular endothelial growth factor alone. This review revealed biomarker modeling that impacts treatment selection. Recent tumor profiling into “angiogenic signature” more sensitive to angiogenic agents versus “immune signature” more likely to achieve the best response with immunotherapy should be validated. Tumor biology features might be more powerful than clinical classification for a tailored treatment approach.

## Take Home Message

The current guidelines recommend the use of immune check inhibitors (anti–PD-1/anti–PD-L1 and anti-CTLA4) alone or in combination with anti–vascular endothelial growth factor (anti-VEGF) therapy in the first-line treatment of renal cell carcinoma, based on a substantial survival benefit as compared with anti-VEGF therapy alone. However, the clinical practice demonstrated that the “one treatment fits all” strategy might not be the best approach. In fact, recent studies showed that the addition of immune check inhibitor (ICI) agents will not benefit equally all patients with metastatic renal cell carcinoma, and some still respond either equally to or better than anti-VEGF alone. Beyond the clinical stratification model routinely used in clinical practice, herein we reported biomarker modeling that has an impact on treatment selection. Recent tumor profiling into the “angiogenic signature” more sensitive to tyrosine-kinase inhibitors versus the “immune signature” more likely to achieve the best response with ICIs could impact patients if used in treatment allocation. Tumor biology features might be more powerful than clinical classification for a tailored treatment.

## 1 Introduction

Approximately 85% of kidney tumors are renal cell carcinoma (RCC), and approximately 70% are of clear cell histology [1], [2]. Patients with organ-confined disease harbor excellent 5-yr survival rate exceeding 92.5%, while only 12% patients with metastatic RCC (mRCC) are alive at 5 yr [3].

For more than a decade, anti–vascular endothelial growth factor (VEGF) agents were the cornerstone of the management of mRCC. Recently, several randomized controlled studies confirmed the life-prolonging effect (compared with sunitinib) of immune check inhibitor (ICI) agents including anti-CTLA4 and anti–PD-L1 given in combination with tyrosine-kinase inhibitors (TKIs) as a first-line treatment in patients with mRCC [4], [5]. Therefore, different guideline panels introduced either a doublet of ICIs (ipilimumab + nivolumab) or TKI/ICI (pembrolizumab + axitinib, avelumab + axitinib, and nivolumab + cabozantinib) combinations in the first line of mRCC [6], [7], [8], [9].

Currently, treatment choice is based only on the International Metastatic RCC Database Consortium (IMDC) risk stratification criteria [10]. Hence, some overlapping may be encountered since intermediate- and poor-risk patients can be offered ICI doublets or ICI/TKI combinations, while favorable risk group patients can only be offered immunotherapy/TKI [6]. To help understand the underlying biology and determine the predictive factors of response to TKIs, and now TKIs and ICIs, many molecular classifications have been reported [11]. Ultimately, predicting disease course and response to treatment would guide the selection of a tailored treatment strategy for every patient in a personalized approach [12].

Herein, our objective was to perform a systematic review of the different molecular classification models reported in the first-line treatment of mRCC and discuss the awaited clinical implications.

## 2 Evidence acquisition

Embase and Medline databases as well as European Society for Medical Oncology (ESMO)/American Society of Clinical Oncology (ASCO) conference proceedings were searched from 2000 and 2021 to identify reports of interest according to the guidelines of “the Preferred Reporting Items for Systematic Reviews and Meta-analyses” (PRISMA) statement [13]. The following MESH search terms were used alone or in combination: “renal cell carcinoma,” “kidney cancer,” “genomics,” “transcriptomics,” “therapeutics,” “molecular classification,” “biomarkers,” “precision medicine,” “immune check inhibitors,” “tyrosine-kinase inhibitors,” and “anti-VEGF.”

According to the PICO framework, inclusion criteria comprised randomized and nonrandomized controlled clinical trials that included patients treated in the first line of metastatic setting, metastatic clear cell renal cell carcinoma (ccRCC) histology, biological modeling, and survival outcomes.

**Fig. 1:**
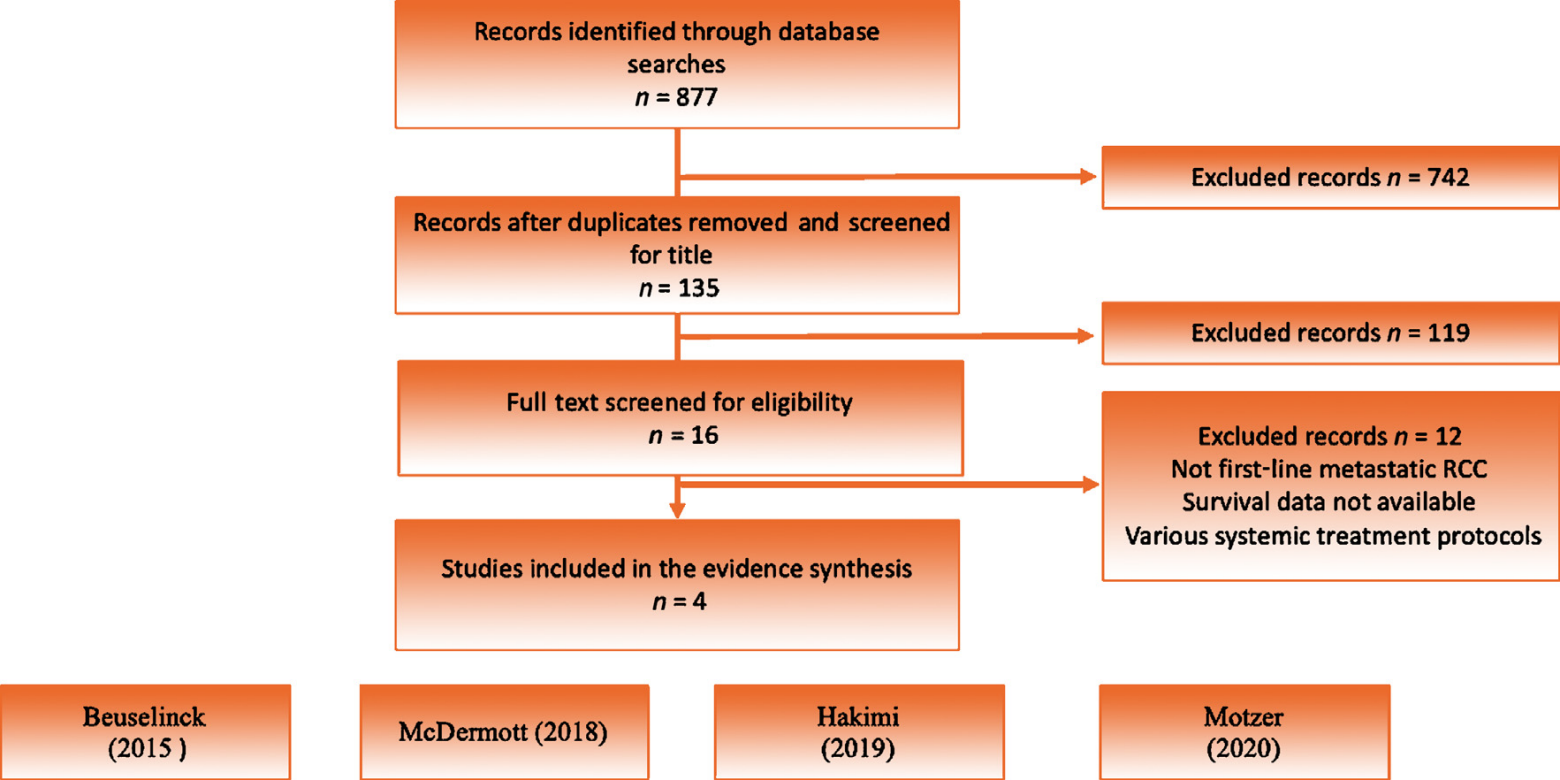
Flowchart of evidence acquisition and search strategy. RCC = renal cell carcinoma.

To ensure consistency with the inclusion criteria, titles and abstracts were reviewed by two authors (I.O. and Z.K.). Articles retained after this first screening were then studied thoroughly (Fig. 1). Given the noncomparative design of the identified studies, evidence synthesis was performed in a descriptive and narrative manner.

## 3 Evidence synthesis

Four major models have been described [14], [15], [16], [17]. Of these, three were ancillary studies of phase 2 or 3 clinical trials, and the data of one model were acquired retrospectively [14]. The main findings of these classifications are summarized in Table 1. For every included study, detailed protocol and sequencing methodology and laboratory techniques are provided in the Supplementary material.

**Table 1:** Molecular classification, features, and outcomes of metastatic renal cell carcinoma treated in the first-line setting

*Molecular subtypes*					
Beuselinck (2015) [14]	Classification	ccrcc1 (MYC.UP)	ccrcc2 (Classical)	ccrcc3 (Normal like)	ccrcc4 (Immune UP/MYC.UP)
	Features	Stem cell polycomb signature and CpG hypermethylation+ VHL mutation = 46.7% PBRM1 mutation = 46.7%	VHL mutation = 62.5% PBRM1 mutation = 37.5%	Transcriptomic signature close to normal samples VHL mutation = 20% PBRM1 mutation = 20%	Stem cell polycomb signature and CpG hypermethylation++ Th1 oriented TME (PD1high, TNF, IRF families, IFNg IL-12) VHL mutation = 20% PBRM1 mutation = 0%
	Outcomes	Low RR, PFS, OS	Better RR, PFS, OS		Low RR, PFS, OS
		PD = 22% PR/CR = 41%	PD = 3% PR/CR = 53%	PD = 0% PR/CR = 70%	Sarcomatoid features PD = 27%, PR/CR = 21%
*Molecular subtypes*					
McDermott (2018) [15]	Classification	Angio^High^	T_eff_^High^		Myeloid^High^
	Features	High vascular density CD131 high	PD-L1 CD8 T-cell infiltration		IL-6, prostaglandins, and the CXCL8 family MDSCs
	Outcomes	High response de sunitinib	High response de bevacizumab + atezolizumab		Best response to sunitinib Worse response to atezolizumab monotherapy
*Molecular subtypes*					
Hakimi (2019) [16]	Classification	Cluster 1	Cluster 2	Cluster 3	Cluster 4
	Features	Angio^low^ Immune^Low^	Angio^High^ Immune^Low^	Angio^High^ Clearcode34 (ccA^high^) = 89% PBRM1^high^ (54%) PD-L^low^ (30%) Immune^Low^	TP53^high^, BAP1^high^ PBRM1^low^ IFNγ high MYC^high^ PD-L1^high^ (60%) Immune^High^
	Outcomes	Similar OS and PFS in Cluster 1-3 No difference between sunitinib and pazopanib			Worse PFS, OS than 1–3
*Molecular subtype*					
Motzer (2020) [17]	Classification	JAVELIN 101 Angio signature		JAVELIN 101 Immuno signature	
	Features	NRARP, RAMP2, ARHGEF15, VIP NRXN3, KDR, SMAD6, KCNAB1 CALCRL, NOTCH4, AQP1, RAMP3 TEK, FLT1, GATA2, CACNB2 ECSCR, GJA5, ENPP2, CASQ2 PTPRB, TBX2, ATP1A2 CD34, HEY2, EDNRB		CD3G, CD3E, CD8B, THEMIS, TRAT1, GRAP2, CD247 CD2, CD96, PRF1, CD6, IL7R, ITK, GPR18, EOMES, SIT1, NLRC3 CD244, KLRD1, SH2D1A CCL5, XCL2 CST7, GFI1, KCNA3, PSTPIP1	
	Outcomes	JAVELIN Angio^High^ longer PFS with sunitinib No difference with avelumab + axitinib		JAVELIN Immuno^high^ longer PFS with avelumab + axitinib No difference with sunitinib	

CR = complete response; IFN = interferon; IL = interleukin; OS = overall survival; PD = progressive disease; PFS = progression-free survival; PR = partial response; RR = response rate; TME = tumor microenvironment; TNF = tumor necrosis factor; VHL = von Hippel-Lindau.

### 3.1 Beuselinck et al model

A global transcriptome analysis of 53 primary resected ccRCC tumors from patients who developed mRCC and were treated with first-line sunitinib was conducted [14]. Chromosome copy-number aberrations, methylation status, and gene mutations in von Hippel-Lindau and PBRM1 were determined. Molecular data were analyzed in relation with response rate (RR), progression-free survival (PFS), and overall survival (OS). An internal validation study using quantitative reverse transcription polymerase chain reaction (qRT-PCR) was performed on 47 additional ccRCC samples treated within the same setting.

Four subtypes have been described with respect to prognosis and biological behavior (Table 1). The ccrcc2 (classical) and ccrcc3 (normal-like) subtypes showed better RR, PFS, and OS than the ccrcc1 (myc-up) and ccrcc4 (immune-up/myc-up) subtypes. Myc-up tumors showed high levels of stem cell polycomb signature and CpG hypermethylation, while immune-up tumors showed a T-cell helper 1 (Th1) oriented tumor microenvironment harboring high levels of PDA expression and proinflammatory mediators (tumor necrosis factor [TNF], IRF family, interleukin [IL]-12). This molecular classification was initially based on tumors treated with sunitinib and has also been validated in patients treated with pazopanib [18].

### 3.2 McDermott et al (IMmotion150) model

Data were prospectively acquired within the IMmotion150 study, a randomized phase 2 study that evaluated atezolizumab (anti–PD-L1) alone or combined with bevacizumab (anti-VEGF) versus sunitinib in 305 patients with treatment-naïve clear cell mRCC [15].

Multiple analyses including whole-transcriptome profiles (TruSeqRNA), indel calling, and whole-exome sequencing (208 patients with tumors and peripheral blood) were performed. Gene signature profiles were defined as angiogenic (VEGFA, KDR, ESM1, PECAM1, ANGPTL4, and CD34), T-cell effective (CD8A, EOMES, PRF1, IFNG, and CD274), or myeloid inflammation (IL-6, CXCL1, CXCL2, CXCL3, CXCL8, and PTGS2).

Three clusters were identified, showing a distinct response to treatment (Table 1). The angiogenic profile characterized by high vascular density showed the best response to sunitinib. The T-cell effective profile showed high PD1 expression, and CD8 infiltrates had the best outcomes to the atezolizumab and bevacizumab combination, while the myeloid profile was less responsive to atezolizumab monotherapy.

### 3.3 Hakimi et al model

The model was developed after integrated genomic and transcriptomic analyses of patients with clear cell mRCC treated with TKI therapy (sunitinib or pazopanib) within the COMPARZ phase III trial (*N* = 409; *n* = 212 sunitinib, *n* = 197 pazopanib) [16]. The study concluded that pazopanib was noninferior to sunitinib with respect to PFS and OS in the first-line treatment of clear cell mRCC [19].

Immunohistochemistry, whole-genome sequencing (next-generation sequencing) and microarray, and RNA-seq were performed on tumor specimens (*N* = 409; *n* = 212 sunitinib, *n* = 197 pazopanib). Gene signatures included the angiogenesis profile (FLT4, FLT1, VEGFB, ENG, KDR, and BAI) and proinflammatory profile (Macrophage, PDL1, IFNγ, IFNα, inflammatory response, IL-6, and TNFα signaling).

Overall, four clusters have been identified of which three (clusters 1, 2, and 3) showed similar outcomes, while cluster 4 (TP53^high^, BAP1^high^, PBRM1^low^, IFNγ high, MYC^high^, PDL1^high^ 60%, and Immune^High^) had the worse PFS and OS (Table 1). In addition, patients in the IMDC poor-risk group were enriched with cluster 4 (45.7%) compared with clusters 1–3 (Fisher’s exact test, *p* = 0.009).

### 3.4 JAVELIN 101 Renal model

The model was developed after the analyses of tumor samples (*n* = 886, 63% nephrectomy and 37% metastatic sites) of patients included in the JAVELIN 101 Renal trial [17]. This randomized phase 3 trial (NCT02684006) demonstrated prolonged PFS with the combination of avelumab (anti–PD-L1) + axitinib (TKI, and targeting VEGF receptors 1, 2, and 3) versus sunitinib (TKI) in previously untreated mRCC patients with clear cell component [20], [21].

Analyses included whole-exome sequencing, gene expression profiling, and immunohistochemistry. Two major profiles emerged from the gene signature analyses that showed different features and outcomes. The “Renal 101 Immuno” profile comprised regulators of both adaptive and innate immune responses (T cell and natural killer cell), cell trafficking, and inflammation (Table 1). Patients with gene expression higher than the median had longer PFS than those with less than the median expression in the avelumab + axitinib arm (hazard ratio [HR] 0.60; 95% confidence interval [CI] 0.439, 0.834; *p* = 0.0019), but the signature did not differentiate between PFS times in the sunitinib arm (HR 0.89; 95% CI 0.670, 1.172; *p* = 0.3973). Similarly, the “Renal 101 Angio” profile identified a 26-gene angiogenesis that significantly differentiated between PFS values in the sunitinib arm (HR 0.56; 95% CI 0.420, 0.741; *p* < 0.0001) but not in the avelumab + axitinib arm (HR 0.98; 95% CI 0.711, 1.340; *p* = 0.8819).

### 3.5 Discussion

Recently, treatment of mRCC paradigm has shifted from anti-VEGF to anti–PD-1/PD-L1 or anti-CTLA4 agents either alone or in combination with an anti-TKI [6] based on survival benefit in the first-line setting [4], [5]. However, the clinical practice demonstrated that the one treatment fits all strategy might not be the best approach to optimize treatment outcomes. In fact, recent studies showed that the addition of ICI agents will not benefit all mRCC patients equally, and some still respond either equally to or better than TKIs alone [4].

Clinical and basic biological criteria included in the IMDC risk stratification failed to demonstrate a tailored approach for treatment. For example, combination therapy with ipilimumab and nivolumab seemed to offer the best outcomes in patients with mRCC with sarcomatoid features with, an unprecedented, complete response in up to 20% of the patients [22]. In addition, PD-1 expression alone does not seem to impact deeply the response to treatment with anti–PD-1/anti–PD-L1 agents [23]. It is then legitimate to identify biological markers to help in decision-making and patient counseling for the best approach.

Current clinical practice questions the remaining role of TKI agents in the front line of mRCC. Evidence reported here suggests an mRCC biological spectrum: the highly angiogenic and the proinflammatory profile at the borders, and a mixed/normal-like profile in the center of the spectrum. The angiogenic profile included ccrcc2 of the Beuselinck model, clusters 2 and 3 in the Hakimi model, and Angio^High^ in the McDermott model. These tumor groups showed the best response to sunitinib. The proinflammatory/Immune^High^ profile included ccrcc4 in the Beuselinck model, cluster 4 in the Hakimi model, and Teff^High^ in the McDermott model, and was more likely to respond to ICI agents either alone or in combination.

Biological features do not always mirror clinical features such as IMDC classification [24]. Tumor profiling might then explain the controversial outcomes reported in clinical trials. In the Checkmate 214 trial that evaluated nivolumab + ipilimumab versus sunitinib in previously untreated clear cell mRCC patients, ICI agents showed a survival benefit in intermediate and unfavorable but not in favorable IMDC risk groups [4]. In accordance with the data reported by Hakimi et al [16], the proinflammatory/Immune^High^ profile could be more represented in the unfavorable IMDC risk group.

**Fig. 2:**
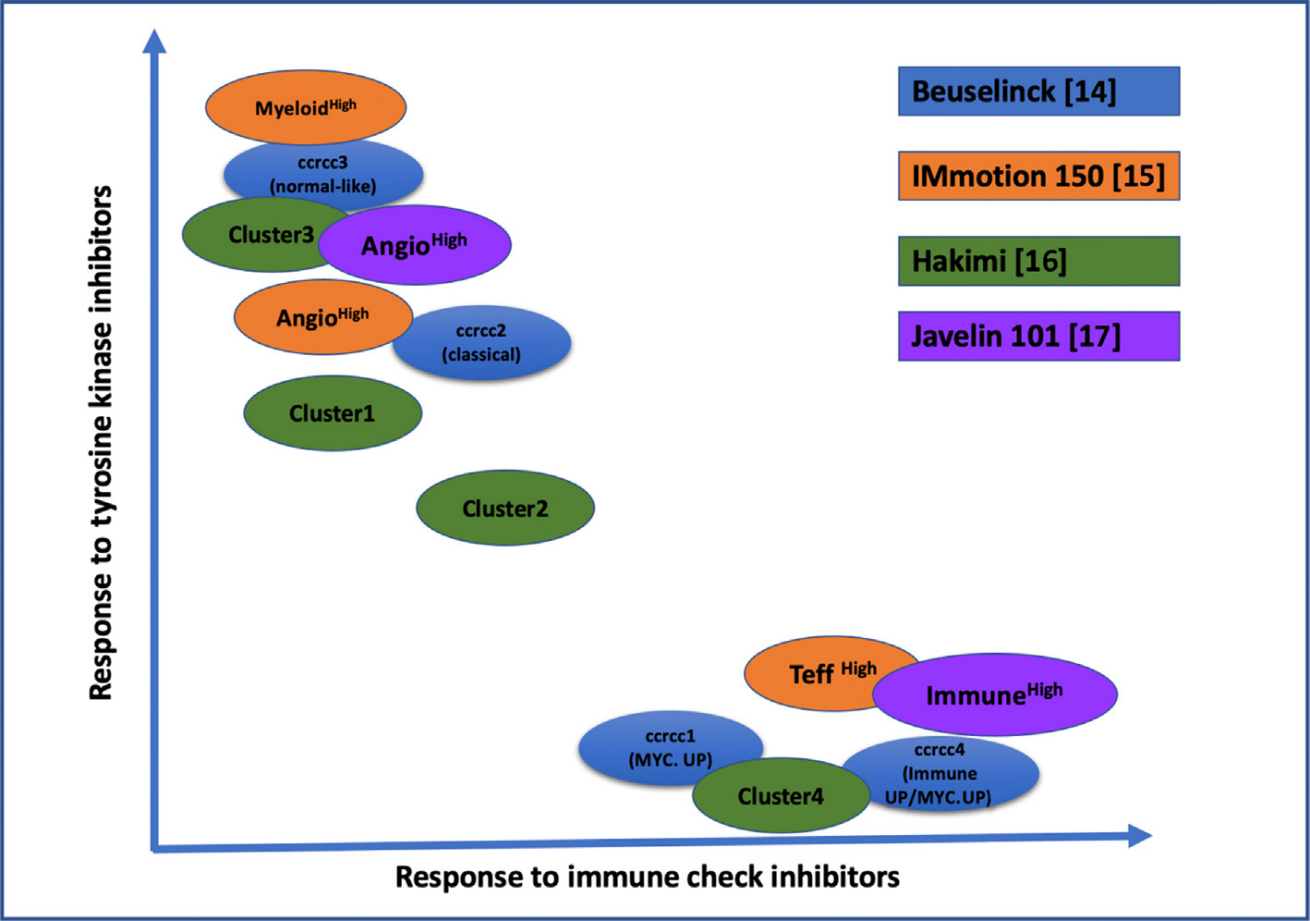
Hypothetic representation of the response to tyrosine kinase inhibitors and immune check inhibitors according to molecular classification in every model.

In the center of the spectrum, ccRCC, cluster 1, and Myeloïd^High^ in the Beuselinck, Hakimi, and McDermott models, respectively, showed good responses to sunitinib as well. This subgroup is very heterogeneous and could be treated by TKI alone or TKI + ICI therapy, although the gold standard in the first-line treatment according to different recommendation panels is currently TKI + ICIs [6], [7]. Based on these findings, we hypothesized a graphic representation of the different models, and their potential responses to current therapies are represented in Figure 2.

Herein, we reported four major molecular classifications that have been reported so far. The Beuselinck model was based on limited specimens (*n* = 53) from patients having mRCC, and survival data were collected retrospectively, which constitute a major limitation [14] unlike the classifications by Hakimi et al [25], which was adjunct to a phase 3 comparative trial with sufficient data quantity/quality-wise. In addition, the Beuselinck model lacked external validation, while the Hakimi model was validated in two external cohorts (Memorial Sloan Kettering Cancer Center [MSKCC] and Beuselinck). Most importantly, TKI alone is not the recommended regimen in the first-line treatment of mRCC anymore, as shown by the Checkmate 214, Keynote 426, and JAVELIN 101 Renal trials [4], [5], [20]. Therefore, the use of the conclusions of these models is currently limited.

The biological models in IMmotion150 and JAVELIN 101 are part of the ancillary biomarker studies adjunct to clinical trials evaluating new drugs including atezolizumab and avelumab in the treatment of kidney cancers [15], [17]. Biomarkers are prospectively collected, and such data are more robust than models built on retrospective data. In fact, the predictive value of JAVELIN 101 signature was validated in independent datasets (the phase 1b JAVELIN Renal 1006 and phase 1b JAVELIN Solid Tumor trials), supporting its relevance and robustness as a potential indicator of PFS benefit following combination immunotherapy [26], [27].

The application of the IMmotion150 gene expression signature (GES) to the JAVELIN Renal 101 dataset enriched PFS in the sunitinib arm but had no effect on the combination arm. Renal 101Angio was significantly associated with prolonged PFS in the sunitinib arm, but did not differentiate between PFS values in the combination arm. Despite both studies using sunitinib as a comparator, IMmotion Angio^High^ and JAVELIN Angio had limited overlap with published GESs: only CD34 and KDR are present in both the JAVELIN Renal 101 Angio and the IMmotion150 angiogenesis GES, and only ECSCR, KDR, PTPRB, and TEK are present in both the JAVELIN Renal 101 Angio and an angiogenesis core signature identified in multiple tumor types [15], [17], [28].

The failure of the JAVELIN 101 trial to demonstrate an OS benefit could limit the impact and use of this signature. Therefore, these GESs were evaluated in the Checkmate 214 trial data set and presented recently [29]. The trial demonstrated the benefit of nivolumab (anti–PD-1) + ipilimumab (anti-CTLA4) in prolonging OS in the first-line setting in IMDC intermediate/high-risk mRCC patients [4]. While the Angio^High^ score (as per IMmotion150) was significantly associated with improved PFS within the sunitinib arm, no other observed significant differences were observed between the remaining GESs. Specifically, OS in patients treated with nivolumab and ipilimumab was similar regardless of the gene signature profile (as per IMmotion150 or JAVELIN 101) [29]. The use of an anti-CTLA4 agent in combination with anti–PD-1 in this trial and the percentage of patients with tumor evaluable for testing (109/550 and 104/546 of patients in the nivolumab + ipilimumab and sunitinib arms, respectively) could be the potential issues for the failure of this study to externally validate the previously reported JAVELIN classification.

Trending consensus is emerging toward the use of gene signatures (as per JAVELIN) and dichotomizing the tumors into “immune” and “angiogenic” profiles. However, such classifications should be validated in more extensive datasets.

To be more conclusive, molecular classifications should be tested in clinical trials with new designs. Treatment should be allocated on the basis of tumor biological characteristics and not only on the basis of the clinical risk stratification group such as IMDC. This new design is featured in the BIONIKK trial (NCT02960906) based on the model reported by Beuselinck et al [14]. This model revealed four groups of patients (ccrcc1 to ccrcc4) with distinct tumor microenvironment composition and distinct outcomes with sunitinib: ccrcc1 “immune-low” and ccrcc4 “immune-high” tumors were associated with the poorest outcome, and ccrcc2 “angio-high” and ccrcc3 “normal-like” tumors were associated with the best outcomes [14]. Consequently, a 35-gene signature (frozen samples, qRT-PCR) was constructed to classify patient by patient in the four groups [30].

Bionikk is a phase 2 trial that hypothesized that nivolumab alone should provide good outcomes in ccrcc4, nivolumab + ipilimumab combination should be necessary to improve outcomes in ccrcc1, and TKI (sunitinib or pazopanib) should provide good outcomes in ccrcc2 and ccrcc3. Therefore, ccrcc1,4 and ccrcc2,3 patients were randomized to receive nivolumab versus nivolumab + ipilimumab and nivolumab + ipilimumab versus TKI, respectively [31]. The primary endpoint was objective response rate (ORR) per treatment and group. The secondary endpoints included PFS, OS, and tolerability. Interestingly, there was no correlation between ccrcc1–4 and IMDC risk groups (*p* = 0.14). In addition, ORR doubled with nivolumab alone in patients with ccrcc4 tumors as compared with ccrcc1 tumors with durable responses. The poor prognosis of these highly infiltrated tumors seemed to be reversed by anti–PD-1 agents. In ccrcc1 tumors, combination of ipilimumab and nivolumab was needed to ensure the best outcome. Finally, ccrcc2 tumors showed a very high RR (53.8%) and nonreached median PFS after 16 mo of follow-up [24]. The ORR to sunitinib historically ranged between 27% and 35% when patients were stratified according to the MSKCC and IMDC models [4], [5], [32]. This first-in-class biomarker-based trial provided a preliminary insight into differential responses when treatment is allocated based on tumor biology. The Bionikk trial could be the first trial to assess the power of a biological model to predict outcomes because patients were randomized to receive treatment based on the biological tumor group. The results of this trial are preliminary and survival data are not mature [24].

Despite its appealing aspects, the model has some limitations. First, unlike the other reported classifications, the molecular subtypes reported by Beuselinck et al [14] have been developed based on retrospective data in a limited set of patients. This model has not yet been validated prospectively and externally. Second, the feasibility of such an approach outside of a clinical trial remains questionable. Of note, determination of the molecular group (qRT-PCR + gene signature) was performed within 15 d after tumor biopsy in this experienced and trained platform, which could be considered an acceptable delay before treatment allocation.

Another drawback of the current molecular modeling is related to the tumor specimen. Transcriptomic data were generated from the analyses of tumors harvested during surgical excision (nephrectomy). The delay between surgery and metastatic progression is variable, and tumor characteristics might change during evolution. After exome sequencing, chromosome aberration analysis, and ploidy profiling on multiple spatially separated samples obtained from primary RCC and associated metastatic sites, Gerlinger et al [33] reported different types of mutations. Some mutations are shared between primary tumors or metastatic sites only. Others are ubiquitous (primary and metastases) or private (unique). Although adding complexity, this distinct pattern of metastatic evolution and the spatiotemporal branched mutations have recently been considered a major breakthrough in the understanding of RCC biology [12]. Another alternative to tackle the quality of the harvested tumor specimen is the use of liquid biopsy as an alternative in this setting [34].

Finally, beyond gene signatures and transcriptomic analyses, exploring tumor biology to tailor treatment should include other aspects including HLA variations, tumor mutational burden, gastrointestinal microbiome, and tumor microenvironment [23]. External validation is mandatory for every tool before its use in clinical practice.

## 4 Conclusions

Recent studies showed that the use of molecular classification as a predictive tool in the treatment of mRCC is promising. Gene signatures are gaining popularity, and biomarker analyses are now systematically included in phase 3 trials. Recent tumor profiling into “angiogenic signature” more sensitive to TKIs versus “immune signature” more likely to achieve the best response with ICIs should be validated before routine use in clinical practice. Biology-based clinical trials for treatment allocation could be the new design for the ultimate validation.

  ***Author contributions*:** Idir Ouzaid had full access to all the data in the study and takes responsibility for the integrity of the data and the accuracy of the data analysis.

  *Study concept and design*: Ouzaid, Rioux-Leclercq, Khene, Bensalah, Kammerer-Jacquet.

*Acquisition of data*: Ouzaid, Khene, Kammerer-Jacquet.

*Analysis and interpretation of data*: Ouzaid, Rioux-Leclercq, Khene, Bensalah, Kammerer-Jacquet.

*Drafting of the manuscript*: Ouzaid.

*Critical revision of the manuscript for important intellectual content*: Rioux-Leclercq, Bensalah, Kammerer-Jacquet.

*Statistical analysis*: None.

*Obtaining funding*: None.

*Administrative, technical, or material support*: None.

*Supervision*: Rioux-Leclercq, Kammerer-Jacquet.

*Other*: None.

  ***Financial disclosures:*** Idir Ouzaid certifies that all conflicts of interest, including specific financial interests and relationships and affiliations relevant to the subject matter or materials discussed in the manuscript (eg, employment/affiliation, grants or funding, consultancies, honoraria, stock ownership or options, expert testimony, royalties, or patents filed, received, or pending), are the following: None.

  ***Funding/Support and role of the sponsor*:** None.
