# Supplementary material for: Can Molecular Classifications Help Tailor First-line Treatment of Metastatic Renal Cell Carcinoma? A Systematic Review of Available Models
Source: Eur Urol Open Sci. 2022 Dec 15;47:12–9. doi: 10.1016/j.euros.2022.11.006 (PMC9789383; doi:10.1016/j.euros.2022.11.006)
Supplement: Supplementary Data 1 [file mmc1.docx]

**Supplementary material**

**Summary of the reported models**

| Study | Beuselinck (2015) | | | |
| --- | --- | --- | --- | --- |
| Patients | 53 | | | |
| Clinical trial | None | | | |
| Validation | Internal validation on 47 patients | | | |
| Treatment | First-line Sunitinib | | | |
| Outcomes | RR, PFS, OS | | | |
| Analyses | -Global transcriptome analysis  -Copy-number  aberrations, methylation status, and  -Gene mutations in VHL/PBRM1 | | | |
| Classification | ccrcc1  (MYC.UP) | ccrcc2  (Classical) | ccrcc3  (Normal like) | ccrcc4  (Immune UP/MYC. UP) |
| Molecular and biological characteristics | Stem cell poly- comb signature and CpG hypermethylation+  VHL mutation =46.7%  PBRM1 mutation=46.7% | VHL mutation =62.5%  PBRM1 mutation=37.5% | Transcriptomic signature close to normal samples  VHL mutation =20%  PBRM1 mutation=20% | Stem cell poly- comb signature and CpG hypermethylation++  Th1 oriented TME (PD1high, TNF, IRF families, IFNg IL12)  VHL mutation =20%  PBRM1 mutation=0% |
| Clinical outcomes | Low RR, PFS, OS  PD=22%  PR/CR=41% | Better  RR, PFS, OS  PD=3%  PR/CR=53% | Better  RR, PFS, OS  PD=0%  PR/CR= 70% | Low RR, PFS, OS  Sarcomatoid features  PD=27%  PR/CR= 21% |

RR: Response Rate

PFS: Progression-Free Survival

OS: Overall Survival

TME : tumor microenvironment

PD: Progressive disease

| Study | McDermott (2018) | | |
| --- | --- | --- | --- |
| Patients | 305 | | |
| Clinical trial | Phase II (Immotion 150) | | |
| Validation | None | | |
| Treatment | bevacizumab + atezolizumab vs sunitinib | | |
| Outcomes | PFS, RR in the ITT and the PDL1 + (PD-L1 on ≥ 1% of IC by IHC) population | | |
| Analyses | - Gene expression  -Whole-transcriptome profiles (TruSeq RNA)  -Gene signatures:   - Angio: VEGFA, KDR, ESM1, PECAM1, ANGPTL4, and CD34; - T_eff_: CD8A, EOMES, PRF1, IFNG, and CD274; - Myeloid inflammation: IL-6, CXCL1, CXCL2, CXCL3, CXCL8, and PTGS2   - Indels calling  - Whole-exome sequencing (208 patients tumors and peripheral blood) | | |
| Classification | Angio^High^ | T_eff_ ^High^ | Myeloid^High^ |
| Molecular and biological characteristics | - High vascular density  - CD131 high | -PDL1  -CD8 T-cell infiltration | -IL-6, prostaglandins, and the CXCL8 family  -MDSCs |
| Clinical outcomes | -High response de sunitinib | -High response de Bevacizumab + atezolizumab | - Best response to sunitinib  - Worse response to Atezolizumab monotherapy |

MDSC: Myeloid derived suppressive cells

| Study | Hakimi (2019) | | | |
| --- | --- | --- | --- | --- |
| Patients | 409 ( n= 212 sunitinib, n=197 pazopanib) | | | |
| Clinical trial | Phase III (Comparz) | | | |
| Validation | Beuselinck and MSKCC validation cohorts | | | |
| Treatment | Pazopanib vs sunitinib | | | |
| Outcomes | OS, PFS | | | |
| Analyses | -Immunohistochemistry  -Whole genome sequencing (Next generation Seq)  -Microarray and RNA-seq -Gene signatures:  - Angiogenesis profile : FLT4, FLT1, VEGFB, ENG, KDR, and BAI  - Proinflammatory profile : Macrophage, PDL1, IFNγ, IFNα, inflammatory  response, IL6, and TNFα signaling | | | |
| Classification | Cluster 1 | Cluster 2 | Cluster 3 | Cluster 4 |
| Molecular and biological characteristics | -Angio^low^  -Immune^Low^ | -Angio^High^  Immune ^Low^ | - Angio^high^  - Clearcode34(ccA^high^) =89%  - PBRM1^high^ (54%)  - PDL ^low^ (30%)  -Immune^Low^ | -TP53 ^high^, BAP1^high^  -PBRM1^low^  -IFNγ high  -MYC^high^  -PDL1^high^ (60%)  -Immune^High^ |
| Clinical outcomes | - Similar OS and PFS in Cluster 1-3  - No difference between Sunitinib and Pazopanib | | | Worse PFS, OS than 1-3 |

| Study | Javelin Renal 101 (2020) | |
| --- | --- | --- |
| Patients | 886 | |
| Clinical trial | Phase III | |
| Validation | Yes | |
| Treatment | avelumab+ axitinib vs sunitinib | |
| Outcomes | PFS, ORR, | |
| Analyses | - IHC (PDL1, CD8+)  - Gene expression  -Whole-transcriptome profiles (TruSeq RNA)  -Gene Signatures  -HLA typing  -Whole genome sequencing | |
| Classification | Javelin 101 Angio Signature | Javelin 101 Immuno Signature |
| Molecular and biological characteristics | NRARP, RAMP2, ARHGEF15, VIP  NRXN3, KDR, SMAD6, KCNAB1  CALCRL, NOTCH4, AQP1, RAMP3  TEK, FLT1, GATA2, CACNB2  ECSCR , GJA5, ENPP2, CASQ2  PTPRB, TBX2, ATP1A2  CD34, HEY2, EDNRB | CD3G, CD3E, CD8B, THEMIS, TRAT1, GRAP2, CD247  CD2, CD96, PRF1, CD6, IL7R, ITK, GPR18, EOMES, SIT1, NLRC3  CD244, KLRD1, SH2D1A  CCL5, XCL2  CST7, GFI1, KCNA3, PSTPIP1 |
| Clinical outcomes | longer PFS with sunitinib  No difference with Avelumab+axitinib | Longer PFS with Avelumab+axitinib  No difference with sunitinib |
